# *Campylobacter jejuni* and casein hydrolysate addition: Impact on poultry *in vitro* cecal microbiota and metabolome

**DOI:** 10.1371/journal.pone.0303856

**Published:** 2024-05-24

**Authors:** E. G. Olson, D. K. Dittoe, C. C. Chatman, E. L.-W. Majumder, S. C. Ricke

**Affiliations:** 1 Department of Animal and Dairy Sciences, Meat Science and Animal Biologics Discovery Program, University of Wisconsin-Madison, Madison, Wisconsin, United States of America; 2 Department of Animal Science, University of Wyoming, Laramie, Wyoming, United States of America; 3 Department of Bacteriology, University of Wisconsin-Madison, Madison, Wisconsin, United States of America; Tokat Gaziosmanpaşa University: Tokat Gaziosmanpasa Universitesi, TURKEY

## Abstract

This study investigates the impact of casein hydrolysates on the poultry ceca inoculated with *Campylobacter* focusing on microbial molecular preferences for different protein sources in the presence of *Campylobacter jejuni*. Three casein sources (intact casein (IN), casein enzyme hydrolysate (EH), and casein acid hydrolysate (AH)) were introduced to cecal contents in combination with inoculated *C*. *jejuni* in an *in vitro* model system incubated for 48 h at 42°C under microaerophilic conditions. Samples were collected at 0, 24, and 48 h. Genomic DNA was extracted and amplified using custom dual-indexed primers, followed by sequencing on an Illumina MiSeq platform. The obtained sequencing data were then analyzed via QIIME2-2021.11. Metabolite extracts were analyzed with ultra-high-performance liquid orbitrap chromatography-mass spectrometry (UHPLC-MS). Statistical analysis of metabolites was conducted using MetaboAnalyst 5.0, while functional analysis was performed using Mummichog 2.0 with a significance threshold set at P < 0.00001. DNA sequencing and metabolomic analyses revealed that *C*. *jejuni* was most abundant in the EH group. Microbial diversity and richness improved in casein supplemented groups, with core microbial differences observed, compared to non-supplemented groups. Vitamin B-associated metabolites significantly increased in the supplemented groups, displaying distinct patterns in vitamin B6 and B9 metabolism between EH and AH groups (P < 0.05). *Faecalibacterium* and *Phascolarctobacterium* were associated with AH and EH groups, respectively. These findings suggest microbial interactions in the presence of *C*. *jejuni* and casein supplementation are influenced by microbial community preferences for casein hydrolysates impacting B vitamin production and shaping competitive dynamics within the cecal microbial community. These findings underscore the potential of nutritional interventions to modulate the poultry GIT microbiota for improved health outcomes.

## Introduction

The intricate relationship between diet and gut microbiota has garnered significant attention in recent years due to profound impacts on overall health. Over the past two decades, there has been substantial growth in the utilization of enzymes in poultry feed, as noted by Bedford and Cowieson [1]. Exogenous enzymes used in poultry feed can influence the composition, size, and abundance of microbial communities within the gastrointestinal tract (GIT). They can also alter the site of nutrient digestion within the GIT, impacting nutrient availability to the microbiome and influencing the balance between beneficial bacteria and pathogens [1].

Exogenous enzymes can alter microbial communities in the GIT by shifting digestion to the anterior compartments and limiting nutrient availability to the posterior GIT microbiome. They enhance intestinal mucin integrity, extend feed retention, reduce inflammation, and boost immune response [1]. Exogenous enzymes play a crucial role in minimizing variability among feed ingredients, thereby boosting feed digestibility, and maximizing nutrient absorption [2]. This, in turn, reduces digesta viscosity. Changes in the viscosity of diets can influence bacterial growth, potentially reducing the competition between the host organism and bacteria for nutrients [2]. Thus, the strategic use of enzyme combinations to target specific substrates in feed, which synergize with endogenous enzymes, has become highly valuable in the poultry industry. Aloo and Oh and Martinez-Lopez et al. highlight the role of hydrolysates and peptides in balancing GIT bacteria [3,4]. Aloo and Oh’s review explores how peptides and hydrolyzed proteins shape GIT microbiota, impacting digestion and growth rate [3]. Host health and pathogen presence affect dietary ingredient quality [1]. Inclusion of protease, phytase, and carbohydrates alters nutrient digestion sites, reducing substrates for putrefactive organisms and increasing availability for beneficial fermentative organisms in lower GIT compartments, potentially reducing pathogen proliferation, such as *Campylobacter* that rely on peptides and amino acids for growth [5–7]. Genes involved in the breakdown of peptides and amino acids are notably more abundant in vigorously colonizing strains compared to weakly colonizing ones [8]. This suggests that the pathogenesis of *C*. *jejuni* is influenced by its phenotype, which in turn may be shaped by the dietary environment. Additionally, as the demand for poultry meat rises alongside nitrogen supplementation in diets, it becomes imperative to comprehend the co-occurrence patterns of *C*. *jejuni* with local microbiota and the metabolic effects related to nitrogen sources. *C*. *jejuni* may have developed adaptations to bolster its survival through interactions with local microbiota [9]. Understanding the intricate interplays between dietary nitrogen overload, GIT microbiota, and *Campylobacter* colonization is crucial for tackling challenges in poultry production and ensuring food safety [10–12].

The digestibility of proteins can be influenced by various factors including the protein source and its concentration, dietary composition, processing methods, protein glycation, and oxidation [13]. Although protein digestion in the host varies, most proteins are cleaved into peptides and amino acids [14]. Amino acid composition, including sequences, types, charge, and dimensional arrangements, influences protein digestion. Post-translational alterations and protein interactions with other food particles can impact GIT microbiota, generating diverse peptides that influence microbial communities [14]. Yu et al. found that feeding rats duck egg white led to a higher abundance of *Proteobacteria* and *Peptostreptococcaceae* and a lower abundance of *Lachnospiraceae* in their ceca compared to rats fed hen egg white, preserved egg white, and casein. Peptide profile differences during digestion were attributed to these observed variations [15].

Understanding the intricate interplay among diet, exogenous feed enzymes, and GIT microbiota in poultry production creates opportunities to optimize feed formulations, improve nutrient availability, and potentially reduce harmful pathogens, ultimately boosting the efficiency and sustainability of poultry and livestock farming. Further research on the microbiome’s response to dietary interventions, including protein hydrolysates, can offer valuable insights for maximizing animal health and productivity. This understanding can also lead to more predictable outcomes and strategies to mitigate pathogens such as *Campylobacter*.

In our study, we used an *in vitro* poultry cecal model inoculated with *C*. *jejuni* to investigate how different protein structures—intact casein, hydrolyzed casein, and acid hydrolyzed free amino acids derived from casein -affect *C*. *jejuni* abundance, microbial composition, and functional changes within the cecal community. Our experimental focus was to evaluate the impact of simultaneous *C*. *jejuni* and casein addition, assessing 1) *Campylobacter* presence on the local microbial community and 2) molecular preferences of casein hydrolysates in a microbial community when inoculated with *Campylobacter*. Our primary hypothesis was that *C*. *jejuni* growth would be most abundant in the acid hydrolyzed casein-supplemented group. Considering the high abundance of proteolytic bacteria in poultry GIT, we hypothesized that all three supplemented groups would enhance microbial composition and diversity compared to the non-supplemented groups. Additionally, we hypothesized that casein hydrolysates with lower molecular weights would contribute to greater microbial diversity, influencing metabolome responses related to peptide and amino acid metabolism.

## Materials and methods

### Poultry ceca acquisition

On day of hatch, Ross 308 broilers (Aviagen, Huntsville, AL, USA) were brought from a commercial hatchery (Welps, Bancroft, IA, USA) to the Poultry Research Laboratory at the University of Wisconsin-Madison (Madison, WI, USA). Chickens were raised based on standard industry guidelines and ethical practices. The animal work for the current study was conducted with a University of Wisconsin-Madison Institutional Animal Care and Use Committee approved protocol (protocol A006627) to ensure the humane treatment of the birds. Chickens were allowed free access to water and commercial least-cost formulated, double pelleted, corn-soybean meal manufactured diets (S1 Table). Cecal samples were obtained from six different 56-day-old chickens, which were humanely euthanized using CO_2_. The collection of these samples was carried out individually using tools that had been immersed in alcohol and sterilized by flame. Following collection, the cecal samples were carefully placed in sterile sample bags (VWR, Radnor, PA, USA). They were subsequently transferred to a portable anaerobic box (Mitsubishi Gas Chemical Co, Japan) which contained oxygen-scrubbing sachets to maintain an anaerobic environment. Immediately after harvest, the cecal samples were transferred to an anaerobic chamber with an atmosphere of 90% nitrogen, 5% H2, and 5% CO2 (Coy Laboratory Products, Grass Lake, MI, USA) for further processing.

### Media preparation

The selection of the media for evaluating microbial dynamics was based on the methodology established in the prior study conducted by Olson et al. [16]. To prepare the Anaerobic Dilution Solution (ADS), the following components were used: 0.45 g/L K_2_HPO_4_, 0.45 g/l KH_2_PO_4_, 0.45 g/L (NH_4_)SO_4_, 0.9 g/L NaCl, 0.1875 g/L MgSO_4_-7H_2_O, 0.12 g/L CaCl_2_-2H_2_O, 1 ml/L 0.1% resazurin, 0.05% cysteine-HCl, and 0.4% sodium carbonate [17,18]. Autoclaved ADS was then cooled to room temperature and allowed to equilibrate for 24 hours within an anaerobic chamber (Coy Laboratories, Grass Lake, MI, USA). The chamber was maintained at the same atmosphere as described above, effectively removing all traces of oxygen from the solution.

### Bacterial cultures

A *C*. *jejuni* strain 700819, was obtained from the American Type Culture Collection (ATCC^®^) and cryogenically preserved in 50% glycerol. To ensure purity, *C*. *jejuni* strain was passaged on Charcoal-Cefoperazone-Deoxycholate Agar (mCCDA; Himedia, Mumbai, India) supplemented with 1 mL/L sodium cefoperazone and 200 μL/L amphotericin B through three consecutive cycles. From this purified culture, a single colony was selected and inoculated into 40 mL of Bolton Enrichment Broth (BEB) without antibiotic supplement and then incubated for 48 h at 42°C using an Advanced Anoxomat^™^ III system (Advanced Instruments, Norwood, MA, USA) with a microaerophilic atmosphere (5% O_2_, 10% CO_2_, 85% N_2_). The cultures were subsequently pelleted and resuspended in 400 mL of fresh BEB, followed by incubation for 24 h under the previously described conditions. After the 24 h, the 200 mL of the fresh *C*. *jejuni* cultures were aliquoted to 50 mL conical tubes, pelleted, decanted, and resuspended in 200 mL ADS. A 4 mL *C*. *jejuni* starter culture of 10^6^ CFU/mL was allocated into designated serum bottles after the pre-incubation step described below for a final concentration of 10^5^ colony forming units (CFU)/mL.

### Microaerophilic *in vitro* system for *Campylobacter*

The effect of the treatment was evaluated following a well-established protocol for the *C*. *jejuni* cecal model, as outlined by Olson et al. and Feye et al. [16,19]. Cecal contents were collected from six individual birds within aseptic chambers, then weighed and diluted at a ratio of 1:3000 by mixing 0.1 g of cecal content with 900 μL of ADS solution. This resuspended cecal material was further diluted by adding 1 mL of it to 299 mL of ADS for each respective cecum sample. A 36 mL aliquot of this diluted cecal content was subsequently transferred into each serum bottle, with or without the addition of 0.4 g of ground chicken feed (S1 Table). To create an appropriate environment, the cultures were covered with aluminum foil and placed in Advanced Anoxomat^™^ III containers (Advanced Instruments, Norwood, MA, USA), where a microaerobic atmosphere was established, containing 5% O2, 10% CO2, and 85% N2. Incubation was carried out at 42°C with continuous agitation at 150 revolutions per minute (RPM) for 24 hours. This step served as a pre-adaptation to acclimate the naturally occurring microbiota in poultry ceca to the new environment, following the method described previously [18–20]. On the following day, the serum bottles were returned to the anaerobic chamber.

**Fig 1:**
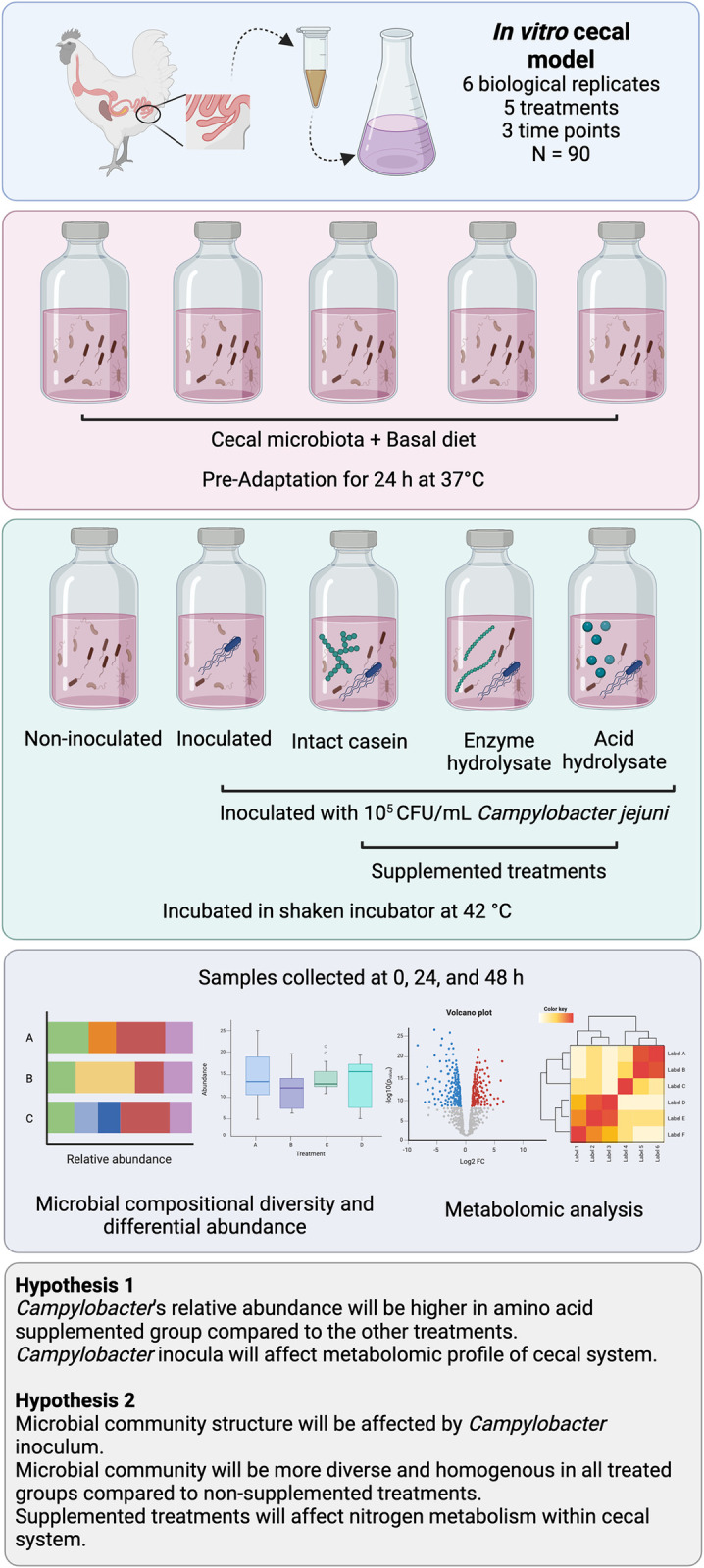
Overview of the experimental design and hypotheses of the current study.

After 24 h of pre-adaption, the serum bottles were moved back into the anaerobic chamber where 4 ml of a freshly prepared 10^6^ cells/mL *C*. *jejuni* culture resuspended in ADS was added to all bottles except the non-inoculated treatment (NI) for a final concentration of 10^5^ CFU/mL. For specific treatments involving casein supplements, 0.1 g of the appropriate vitamin free-casein type (intact (Envigo®, Madison, WI, USA), enzyme hydrolyzed (MilliporeSigma, Burlington, MA, USA), or acid hydrolysate of casein (MilliporeSigma, Burlington, MA, USA) was added to the serum bottles. The study included five distinct treatments: non-inoculated (NI), inoculated with *C*. *jejuni* (IN), and three supplemented groups, intact casein (IC), enzyme hydrolysate (EH), acid hydrolysate (AH), that were all inoculated with *C*. *jejuni*. We opted not to include additional controls for supplemented groups without *Campylobacter*, instead using non-inoculated and inoculated treatments without casein supplementations for comparative analyses with the casein-enriched groups. Because *Campylobacter* naturally inhabits poultry cecal compartments [21], we confirmed the absence of culturable *Campylobacter* in the NI group and confirm the inocula, the 0-hour samples were plated on mCCDA [22]. At 0, 24, and 48 h post-inoculation, duplicate 1 mL samples were taken for microbiome sequencing and metabolomic analysis. The samples were flash-frozen in liquid nitrogen and stored at -80°C until processing. An overview of the procedure is described in Fig 1.

### Microbiota sequencing

DNA was extracted from the aseptically collected aliquots of the microaerobic cultures taken at designated time points (0, 24, 48 h) using Qiagen QIAmp Blood and Tissue Kit (Qiagen, Hilden, Germany) as previously described by Olson et al. [16]. The DNA purity was assessed via a spectrophotometer (Tecan NanoQuant Plate^™^, Mannedorf, Switzerland) and diluted to 10 ng/μL. The paired-end sequencing libraries were arranged by targeting the hypervariable region 4 (V4) of the 16S rRNA gene with PCR primers containing the linker and adapter sequence as described by Olson et al. and Kozich et al. to amplify each DNA sample [16,23]. Sample amplification was verified using gel electrophoresis. PCR products were normalized using a SequalPrep^™^ Normalization kit (Life Technologies, Carlsbad, CA, USA). Five μL of each sample was combined to generate a pooled plate library, and concentration was determined using a KAPPA Library Quantification Kit (Kappa Biosystems, Inc., Wilmington, MA). The library and PhiX control v3 were denatured and diluted to 20 pM in HT1 Buffer and 0.2 N fresh NaOH. The library and PhiX control v3 were further diluted in HT1 buffer to generate a final concentration of 6 pM/L. The resulting library was combined with the diluted PhiX control v3 (20%, v/v), and 600 μL was loaded onto a MiSeq v2 (500 cycles) reagent cartridge (Illumina, San Diego, CA, United States).

### Microbiota bioinformatic analysis

The demultiplexed data were downloaded locally and then transferred to QIIME2 (2021.11) via the Casava1.8 paired-end pipeline [24]. Within QIIME2, the data were denoised and filtered using DADA2, applying the chimera consensus pipeline [25]. To evaluate the diversity of the microbial populations, both alpha and beta diversity analyses were conducted. The QIIME2 phylogeny align-to-tree-mafft-fasttree methodology (q2-phylogeny) was employed for this purpose [26]. For the taxonomic classification of amplicon sequence variants (ASVs), the classify-sklearn tool within QIIME2-2021.11 was utilized in conjunction with the SILVA database (silva-138-99-nb-classifier.qza), employing a 97% confidence threshold [27,28]. Taxonomic data at the genus level were exported from QIIME2 and visualized in JMP Pro 16 (SAS, Cary, North Carolina, USA) with taxa representing less than 1% of the relative abundance being displayed as “other.”

All available metrics related to alpha and beta diversity were computed through QIIME2 diversity core-metrics-phylogeny, with a chosen sampling depth of 6150, retaining 37% (492,000) of features in 80 (95%) samples. The adequacy of this sampling depth was validated by examining alpha rarefaction plots. Additionally, the statistical differences between treatments on the alpha and beta diversities were assessed via q2-diversity. The alpha diversity was analyzed using analysis of variance (ANOVA) to test the interaction of variables via q2-longitudinal [29]. Alpha diversity was assessed for both richness (using the Shannon Diversity Index) and evenness via Pielou’s Evenness [30]. Kruskal-Wallis was used to determine pairwise differences [31]. Differential abundance analysis was performed using ANCOM via q2-composition [32]. Interaction analysis for beta diversity metrics was carried out using permutational multivariate analysis of variance (PERMANOVA) via ADONIS [33]. Beta diversity metrics were evaluated using quantitative indicators, specifically the Bray-Curtis dissimilarity index and the Weighted Unifrac distance matrix [34], employing the Analysis of Similarity (ANOSIM) function which considers the mean population variation and dispersion [35]. Microbiota main effects were considered statistically significant if the main effect exhibited a P < 0.05, and the pairwise effect exhibited a Q < 0.05, with each statistical measurement conducted within the QIIME2-2021.11 pipeline. The Q-value reflected the P-value adjusted for a stringent false discovery rate. Both alpha and beta diversity results were compiled and exported from QIIME2 using the metadata tabulate function. All results were uploaded to JMP Pro 16 where volatility plots, boxplots, and principal coordinate plots were generated.

Core microbiota analysis was conducted using core members and the phyloseq package in RStudio Version 1.3 for five different treatment groups. The core microbiota was defined as ASVs representing at least 5% of the population and detected in 50% of the samples. The Venn diagrams were generated via jvenn [36]. The sequences were submitted to Short Read Archive (PRJNA1018262) under the project title "DNA-Response of Poultry Cecal Microbiota Inoculated with *Campylobacter jejuni*.*"* Detailed sample identification and descriptions are available in the metadata file uploaded to the Short Read Archive managed by the National Center for Biotechnology Information (NCBI).

### Metabolite extraction

As larger proteins do not reach lower GIT compartments [37] and metabolomic changes precede microbial compositional differences [38], our focus was on EH and AH supplemented groups, along with NI and IN treatments at 0 and 24 h, to describe metabolomic profiles (Fig 1). Cell extraction was performed using 0.5 mL of digesta diluent from samples for 0 and 24 h (total samples (N) = 44). Cell lysis was performed using three freeze-thawing cycles which consisted of thawing at room temperature for 10 min followed by a 30 s sonication on ice. A 1 mL aliquot of an acetonitrile:methanol:water (2:2:1 v/v) solvent mixture was added to cell extracts and sonicated for 30 s and stored at -20°C overnight to allow cellular debris and protein to precipitate. The next day samples were centrifuged for 15 min at 13,000 revolutions per minute (rpm) at 4°C. The supernatant was transferred to new Eppendorf tubes (Eppendorf, Hamburg, Germany) and dried for 4 h on a SpeedVac Concentrator (Thermo Scientific Savant DNA 120). Dried samples were then reconstituted in an acetonitrile:water (1:1 v/v) solvent mixture based on the normalized protein concentration of all samples. Normalized volumes were determined using the Bradford method, where the highest protein concentration is equal to 100 μL. Samples were then vortexed for 30 s, sonicated on ice for 10 min, and centrifuged for 15 min at 13,000 RPM at 4°C to remove any residual debris. The metabolite extracts were transferred into autosampler vials with inserts and stored at -80°C until analysis.

### Untargeted metabolomics

Metabolite extracts were analyzed with ultra-high-performance liquid orbitrap chromatography mass spectrometry (UHPLC-MS) (Thermo Scientific Orbitrap Exploris 240 mass spectrometer). A Kinetex Core-Shell 100 Å column C-18 column (1 x 150 mm, 1.7 μm, Phenomenex) was used for separation of metabolites in positive mode. Sample injection volume was 3 μL/min at a flow rate of 0.250 mL/min. Mobile phase A was composed of water with 0.1% formic acid, and mobile phase B was 0.1% in acetonitrile. The gradient began with 5% B from 0–3 min, followed by an increase and held to 95% B until 18 min, followed by a decrease to 5% B at 18.50 min and hold at 5% B until 22 min. Data-dependent acquisition was used for the tandem MS workflow. The MS1 spectra were matched to spectra and compounds in the Human Metabolome Database using MetaboAnalyst 5.0.

MetaboAnalyst 5.0 was used for statistical analysis (P < 0.05). The raw files were converted to mzXML files and centroided (MS-1, orbitrap, positive mode) using ProteoWizard version 3.0 [39]. Pairwise comparisons were performed to investigate potential differences of metabolite features between treatment groups using t-test and volcano plots for visualization of the results. Functional analysis was conducted using Mummichog 2.0 (with a significance threshold of P-value < 0.00001) utilizing the KEGG *Escherichia coli* library within MetaboAnalyst. To identify dysregulated microbial metabolites and metabolic pathways, metabolite features were annotated using Compound Discoverer (Thermo Fisher Scientific, Waltham, MA), and subsequently mapped to the metabolic pathways of representative cecal bacterial members *C*. *jejuni jejuni 51037*, *F*. *prausnitzii* A2-165, and *P*. *faecium* JCM 30894 in BioCyc [37].

## Results

### Casein supplements exerted a significant influence on microbial composition

**Fig 2:**
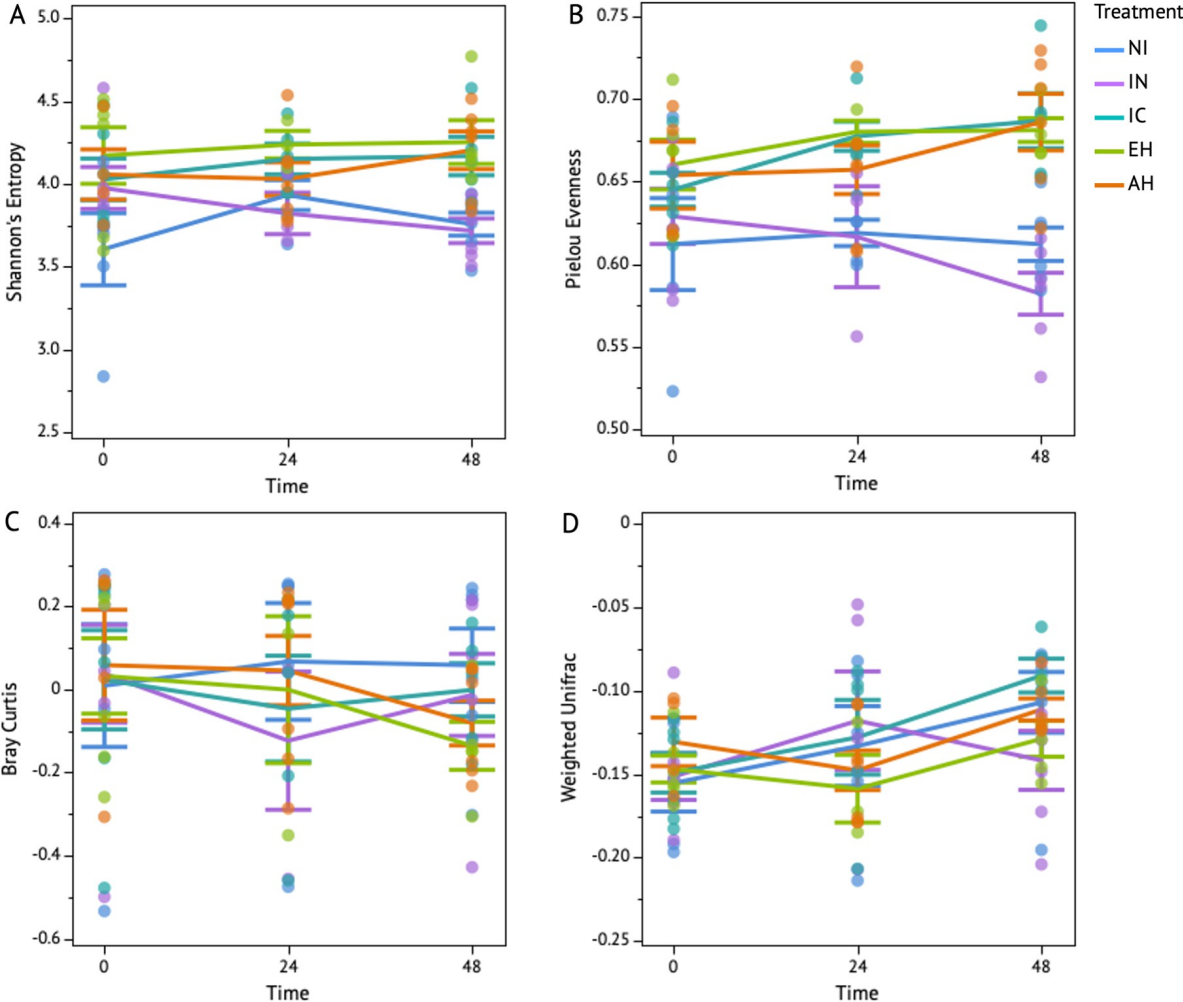
Volatility plots depict the fluctuations in the microbial composition and diversity. **T**he microbial compositional analysis included: Richness (a) and evenness (b) of the microbial community for five treatments (non-inoculated (NI), inoculated (IN), intact casein (IC), enzyme hydrolysate (EH), and acid hydrolysate (AH) across time points (0, 24, and 48 h), utilizing alpha-diversity metrics to capture changes within the samples. Meanwhile, Figure c and d illustrate microbial abundance and phylogenetic compositional changes between the samples, respectively, based on beta-diversity metrics. The whiskers associated with each time point represent the standard deviation for each treatment.

In this study, we investigated the influence of different molecular sizes of casein proteins—namely intact casein, enzyme hydrolysate of casein, and acid hydrolysate of casein—on the cecal microbial composition inoculated with *C*. *jejuni* over a 48-hour period under microaerophilic conditions (total (N) = 84, treatments (k) = 5). To discern potential combined effects of time and treatment on microbial compositions, we employed ANOVA for assessing variable interactions related to evenness and richness parameters within the samples. This revealed there was no significant interaction between treatment and time on microbial richness (ANOVA, Shannon’s Entropy; P > 0.05, S2 Table). However, a trend towards a significant interaction between time and treatment was observed in regard to microbial evenness (ANOVA, Pielou’s Evenness; P = 0.09, S2 Table). When considered individually, treatment drove a significant effect on microbial evenness and richness (P < 0.05, S2 Table). Additionally, when compared to the NI and IN treatments, which exhibited a decline in microbial homogeneity and richness over time, the supplemented groups demonstrated a sustained homogeneity and richness of microbial populations (Fig 2A and 2B). Additionally, ADONIS was utilized to examine phylogenetic diversity and abundance disparities between the samples. Time and treatment exhibited a significant interaction concerning non-phylogenetic-based microbial abundance, as evidenced by the Bray-Curtis analysis (P < 0.05, S3 Table), while no such significance was observed for phylogenetic-based diversity analysis via Weighted Unifrac (P > 0.05, S3 Table). When evaluated individually, treatment demonstrated a significant effect on the phylogenetic composition of microbial communities (P < 0.05, S3 Table). Diverse responses were observed in microbial abundance among the treatment groups. The NI group exhibited stability over time, while both the IN and IC groups initially experienced a decrease in microbial abundance at 24 h, followed by a recovery to their original levels by 48 hours. Conversely, the EH and AH groups demonstrated stability at 24 h but exhibited a decline by the 48-h mark (Fig 2C). Additionally, while the phylogenetic composition of most groups increased with time, the IN treatment exhibited a decreasing trend in phylogenetic composition over time (Fig 2D). These findings reveal that *C*. *jejuni*-inoculated microbial communities, subjected to supplementation with casein hydrolysates, exhibit significant alterations in microbial evenness and richness, impacting abundance dynamics and phylogenetic composition of microbial communities over time.

### Insights from cecal inoculation with *C*. *jejuni* and its minimal impact on microbial community diversity and the metabolome

**Fig 3:**
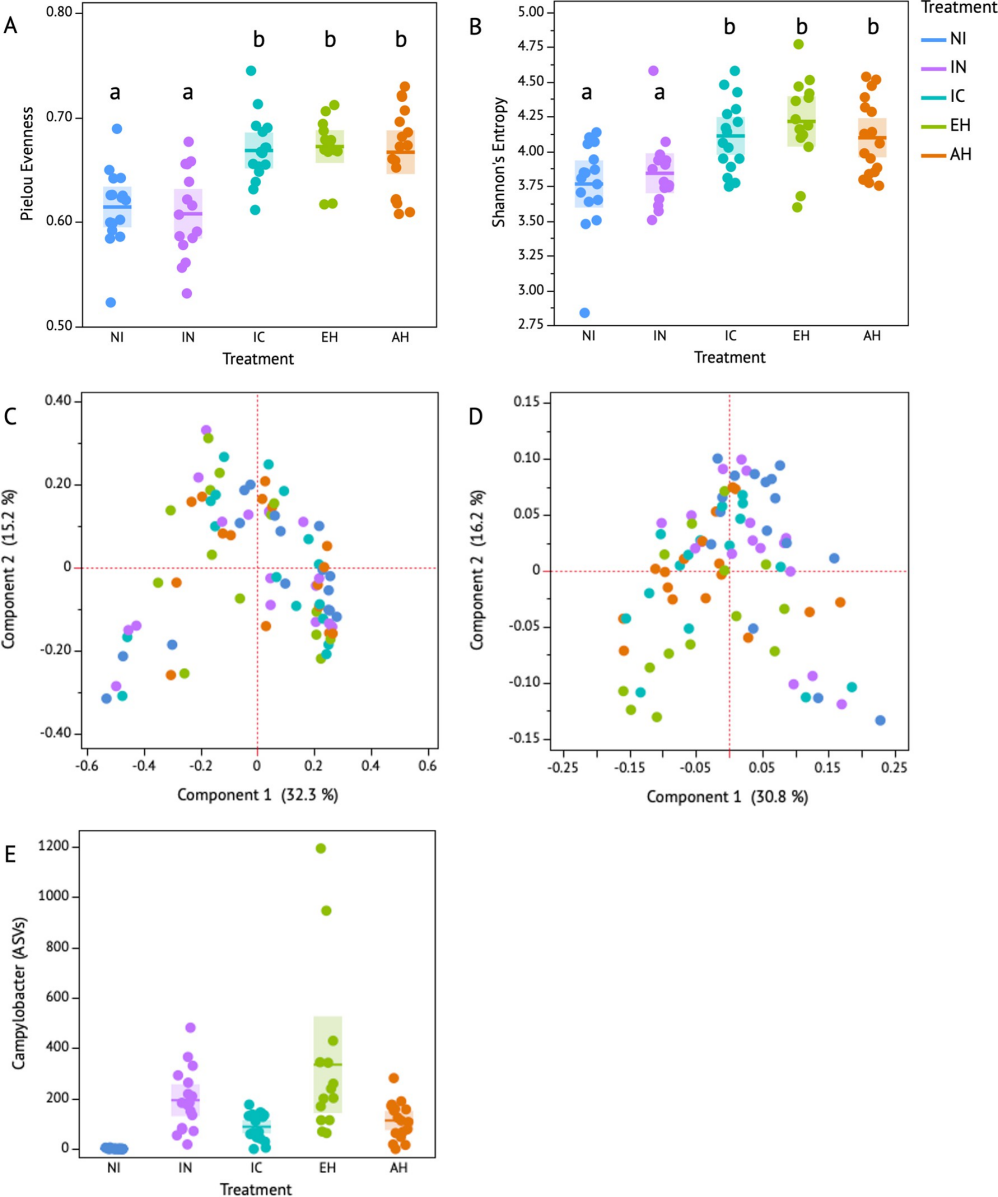
Microbial diversity and composition associated with the supplementation of acid and enzyme hydrolysates, and intact casein. The effect of treatments, non-inoculated (NI), inoculated (IN), intact casein (IC), enzyme hydrolysate (EH), and acid hydrolysate (AH), on alpha diversity, microbial evenness (**a**, Pielou’s Evenness) and richness (**b**, Shannon’s Diversity); beta diversity, differences in abundance (**c**, Bray Curtis) and phylogenetic composition (**d**, Weighted Unifrac); and differentially abundant taxa, ANCOM (**e**), which identified *Campylobacter* as significantly differentially abundant 163 other identified taxa (P < 0.05) where the y-axis denotes *Campylobacter* abundance as amplicon sequencing variants (ASVs). The shaded boxplots represent the standard deviations, and the solid lines represent the mean. Significant differences are denoted by the connecting letters a-b.

Before addressing the main objective of the current study, which focuses on the effects of peptide supplementation on a cecal microbiota inoculated with *C*. *jejuni*, we first aimed to understand the impact of *C*. *jejuni* on the cecal system without casein supplementation. This allowed us to assess if the presence of *C*. *jejuni* alone influences changes in microbial composition and metabolomic-based outputs. The inoculation of cecal contents with *C*. *jejuni* did not drive a significant impact on the microbial evenness and richness when compared to the NI treatment (Pielous’s Evenness, P > 0.05; Shannon’s Entropy, P > 0.05, Fig 3A and 3b; S4 Table). Additionally, neither microbial abundance nor taxonomy were significantly changed in response to the presence of *C*. *jejuni* within the cecal microbial community (Bray-Curtis, P > 0.05, Fig 3C; Weighted Unifrac, P > 0.05, Fig 3D; S5 Table). Significantly, the abundance of C. jejuni stood out from the other 161 identified taxa in this study (ANCOM, P < 0.05). On average, 206 ASVs were associated with *C*. *jejuni* abundance in the IN group, contrasting sharply with the mere 2.5 ASVs observed in the NI group (Fig 3E).

**Fig 4:**
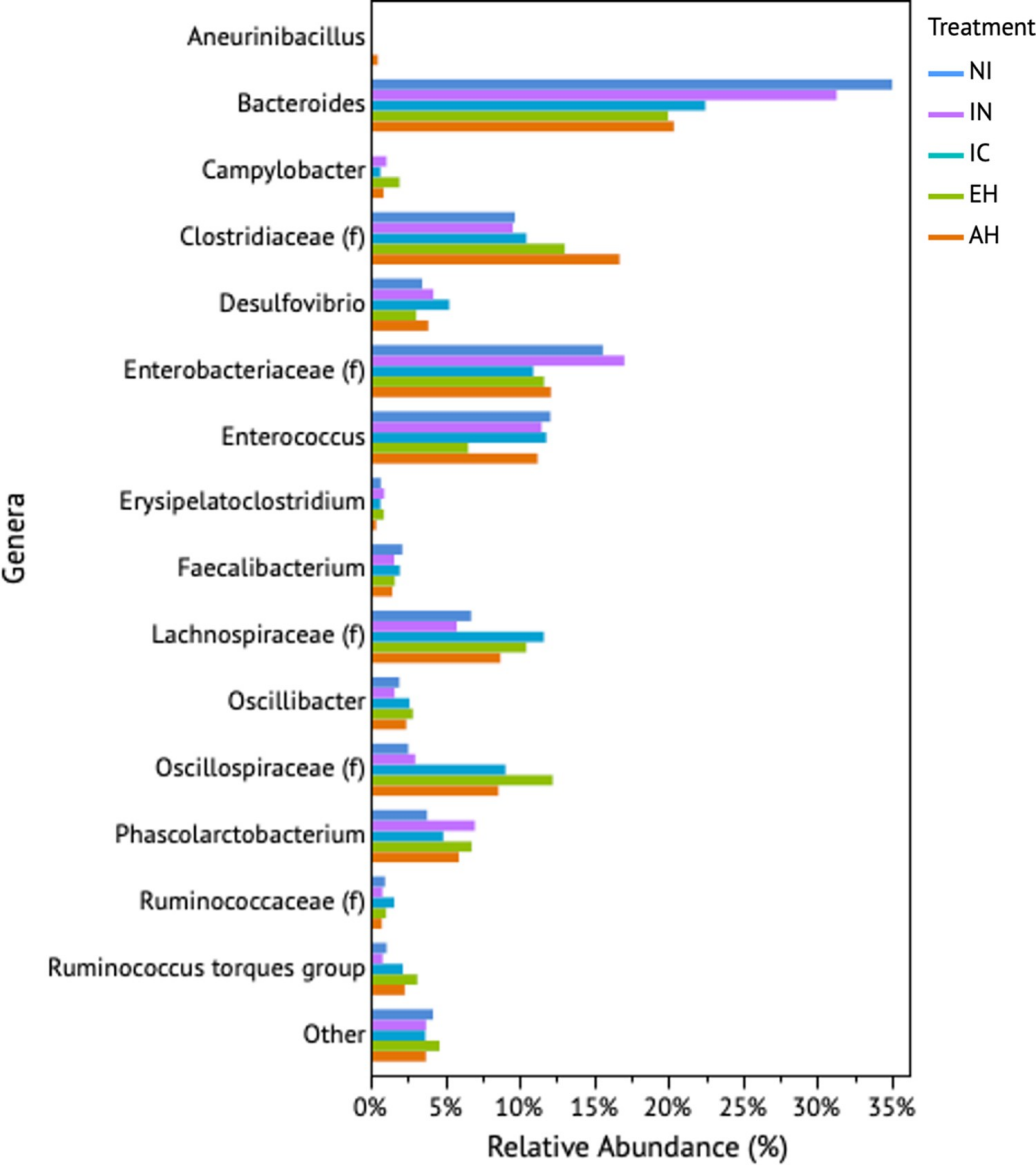
Highly represented genera (>1%) among poultry cecal *in vitro* systems associated with treatments. The treatments consisted of non-inoculated (NI), inoculated (IN), intact casein (IC), enzyme hydrolysate (EH), and acid hydrolysate (AH), used in the current study.

**Fig 5:**
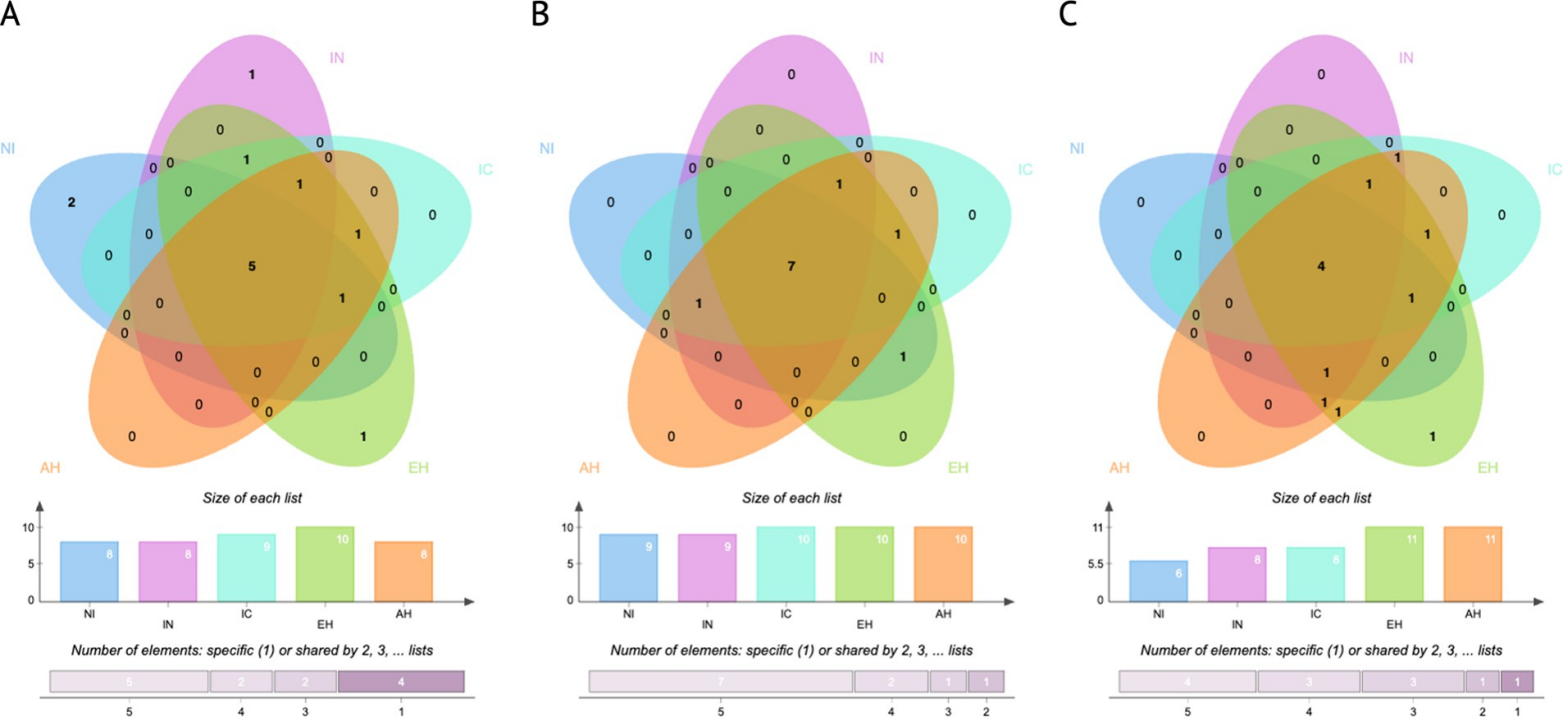
Venn diagram of core microbiota shared between treatments. The treatments in the current study consisted of non-inoculated (NI), inoculated (IN), intact casein (IC), enzyme hydrolysate (EH), and acid hydrolysate (AH), at 0 (**a**), 24 (**b**), and 48 h (**c**).

As shown in Fig 4, among the 161 observed taxa, 15 distinct taxa were identified, with each representing at least 1% of the microbial population associated with the treatments (S6 Table). However, to assess disparities in core microbial populations, we conducted an analysis focusing on core microbial members at each time point within each treatment. Specifically, we considered ASVs that constituted at least 5% of the population and were consistently present in 50% of the samples, thus qualifying as core members of the microbial community. The stringent parameters for core microbiota assessment were established based on the consistent increase in abundance of *C*. *jejuni* observed in the casein-supplemented groups at each time point. Therefore, the conservative core microbial parameters were defined to effectively isolate *C*. *jejuni* from the core microbial population at any given time point. At the onset (0 hours, Fig 5), there were five core microbial members common to both the NI and IN treatments, collectively representing approximately 45% of the total community. Notably, the treatments diverged by three core members, with *Campylobacter* being exclusive to the IN group. Progressing to the 24-h mark, the treatments shared eight common core microbial members, comprising approximately 89% of the total community membership, where the IN treatment uniquely contained *Campylobacter*. The common core microbial community members at 24 h consisted of Enterobacteriaceae, Oscillospiraceae, *Faecalibacterium*, Clostridiaceae, Lachnospiraceae, *Enterococcus*, *Bacteroides*, and *Ruminococcus torques* group. By the 48-h mark, the common core microbial community narrowed to five members, making up 56% of the total population (Fig 5). In this phase, the IN group exclusively featured *Phascolarctobacterium*, *Faecalibacterium*, and *Bacteroides*, while the NI group contained *Oscillibacter* (S7 Table).

Furthermore, the differences in the metabolomic profiles between the treatments were assessed using 2-fold-change of log transformed metabolite expression using a t-test. The effect of *C*. *jejuni* on the cecal microbial community metabolomic output was compared by assessing the pairwise differences for metabolite feature intensity between IN and NI (False Discovery Rate (FDR) < 0.05). We detected no significant differences in the microbial metabolites between IN and NI at 24 h (FDR > 0.05). Ultimately, while *C*. *jejuni* induced a distinct microbial profile marked by its own elevated abundance, the overall microbial diversity and metabolomic composition within the cecal system remained relatively unchanged.

### Microbial molecular preferences for casein: Richness, abundance, and core members in casein hydrolysate-modified microbial communities with insights into metabolomic transformations

The main objective of the current study was to assess molecular preferences for casein in a microbial community inoculated with *C*. *jejuni*. Although not significantly different from each other, casein supplemented groups (IC, EH, and AH) resulted in a significantly higher evenness and richness compared to non-supplemented treatments (IN and IN) (P < 0.05, Q < 0.05; Fig 3A and 3B). Additionally, AH and EH resulted in significantly different microbial abundances relative to NI (Bray-Curtis, P < 0.05, Q < 0.05, Fig 3C). Considering phylogenetic microbial differences, AH, EH, and IC groups drove significantly different microbial compositional profiles than non-modified treatments (Weighted Unifrac, P < 0.05, Q < 0.05, Fig 3D). For the assessment of microbial composition based on differential abundance, ANCOM was employed. *Campylobacter* abundance was differentiated from the 161 identified taxa associated with the five treatments in the current study (ANCOM, P < 0.05; Fig 3E, S8 Table). Moreover, the abundance of *Campylobacter* was notably distinct in the EH group (ANCOM, P < 0.05), leading to an average increase of 371 ASVs (standard error (SE) = 212.9, n = 5), in contrast to AH (124 ASVs, SE = 47.9, n = 5) and IC (90 ASVs, SE = 31, n = 5).

We analyzed the core microbial community (> 5% of population in > 50% of samples) associated with each treatment over time and identified a set of fourteen core microbial members across the different treatment groups (Fig 5, S6 Table). Notably, *Campylobacter* was consistently a part of a core community in all supplemented groups, except for its absence in IC, AH, and IN treatments at the 48-h mark. *Faecalibacterium* was a member of a core community in all supplemented treatments, except for the EH group at both 24 and 48 h. *Lachnospiraceae* was detected in all supplemented groups. Enterococcus represented at least 5% of the population in all groups except NI at the initial 0-h mark. *Bacteroides* represented at least 5% of the population in all groups except IN at the start (0 h). *Oscillospiraceae* was identified as part of a core microbial population in all groups except for NI and AH at the beginning (0 h). *Oscillibacter* was observed in all protein-supplemented groups at 24 and 48 h, while in control groups, it was only present in NI at the 48-h mark. The *Ruminococcus torques* group was identified in all treatment groups at 24 h and in all groups at 48 h, except for IC. Additionally, it was a part of a core community at the 0-h time point in all groups except NI and IN. *Clostridiacea* was consistently present in all treatment groups across all time points. *Desulfovibrio* was part of the core microbial community in EH and AH at the 48-hour time point and in NI at the 0-h mark. *Phascolarctobacterium* was identified as a core microbial member in the EH treatment at 24 and 48 h, in the AH group at 0 and 24 h, and in both NI and IN groups at 0 and 24 h.

In addition, the common core microbial members between the supplemented groups were analyzed. At the 0-hour time point, there were eight common core taxa across the three treatments. One distinct taxon, Oscillospiraceae, was found in IC and EH but was absent in AH. *Erysipelatoclostridium* was exclusively present in EH at 0 hours, distinguishing it from the other supplemented groups. At the 24-hour mark, there were nine common taxa shared among the treatments. Notably, *Phascolarctobacterium* was unique to the EH group, while *Faecalibacterium* was found in both AH and IC groups but not in EH. At the 48-hour time point, there were seven common taxa shared among all supplemented groups. Ruminococcaceae was exclusively found in IC, *Campylobacter* in EH, and *Faecalibacterium* in AH. Additionally, *Desulfovibrio*, *Phascolarctobacterium*, and the *Ruminococcus* torques group were shared between the EH and AH treatments.

A detailed description of all the metabolites can be found in S9 Table. To gain insights into the fundamental metabolomic distinctions between the AH and EH supplemented groups, we conducted a comparative analysis between these two groups, with the IN treatment serving as the reference. Metabolites associated with peptide supplementations were assessed by comparing metabolite expression between AH versus IN and EH versus IN at 0 h (FDR < 0.05). There were 2,979 insignificantly affected metabolites in AH compared to IN at 0 h. There were 150 significantly down-regulated metabolites in AH and 1,687 significantly up-regulated metabolites in AH compared to IN at 0 h. There were 2,456 insignificantly affected metabolites in EH compared to IN at 0 h. There were 186 significantly down-regulated metabolites and 2,340 significantly up-regulated metabolites in EH compared to IN at 0 h.

We focused on EH and AH supplemented groups, along with NI and IN treatments at 0 and 24 hours, to examine metabolomic profiles, considering that larger proteins do not reach lower GIT compartments [37], and metabolome changes precede microbial compositional differences [38]. Thus, metabolism by cecal microbiota of different hydrolysates of casein were assessed by comparing metabolite feature intensity between EH at 24 and 0 h and AH at 24 and 0 h (FDR < 0.05). There were 3,480 insignificantly affected metabolites in AH compared to 0 h. There were 885 significantly down-regulated metabolites in AH and 414 significantly up-regulated metabolites in AH compared to 0 h. There were 4,439 insignificantly affected metabolites in EH compared to 0 h. There were 391 significantly down-regulated metabolites and 136 significantly up-regulated metabolites in EH compared to 0 h. The metabolomic differences between EH and AH were assessed by comparing metabolite expression between EH and AH at 24 h (FDR < 0.05). There were 3,722 insignificantly affected metabolites. There were 364 significantly up-regulated metabolites in AH and 892 significantly up-regulated metabolites in EH at 24 h.

In the EH supplemented treatment group, an intriguing finding emerged: *Campylobacter* stood out as the most abundant genus. This distinct prevalence of *Campylobacter* in the EH-modified group highlights a noteworthy microbial response to the supplementation, adding a significant layer to the overall findings of the study. Notably, AH and EH modifications resulted in distinct microbial abundances and compositional profile compared to the non-modified group. Across treatment groups and time, fourteen core taxa were observed. However, the common microbial members between supplemented groups showcased temporal and treatment-specific variations. Metabolome analysis revealed substantial differences in metabolite feature intensity, emphasizing peptide supplementation’s molecular impact on the cecal microbiota.

### Impact of casein supplementation and *C*. *jejuni* inoculation on vitamin B metabolism in the cecal system

**Fig 6:**
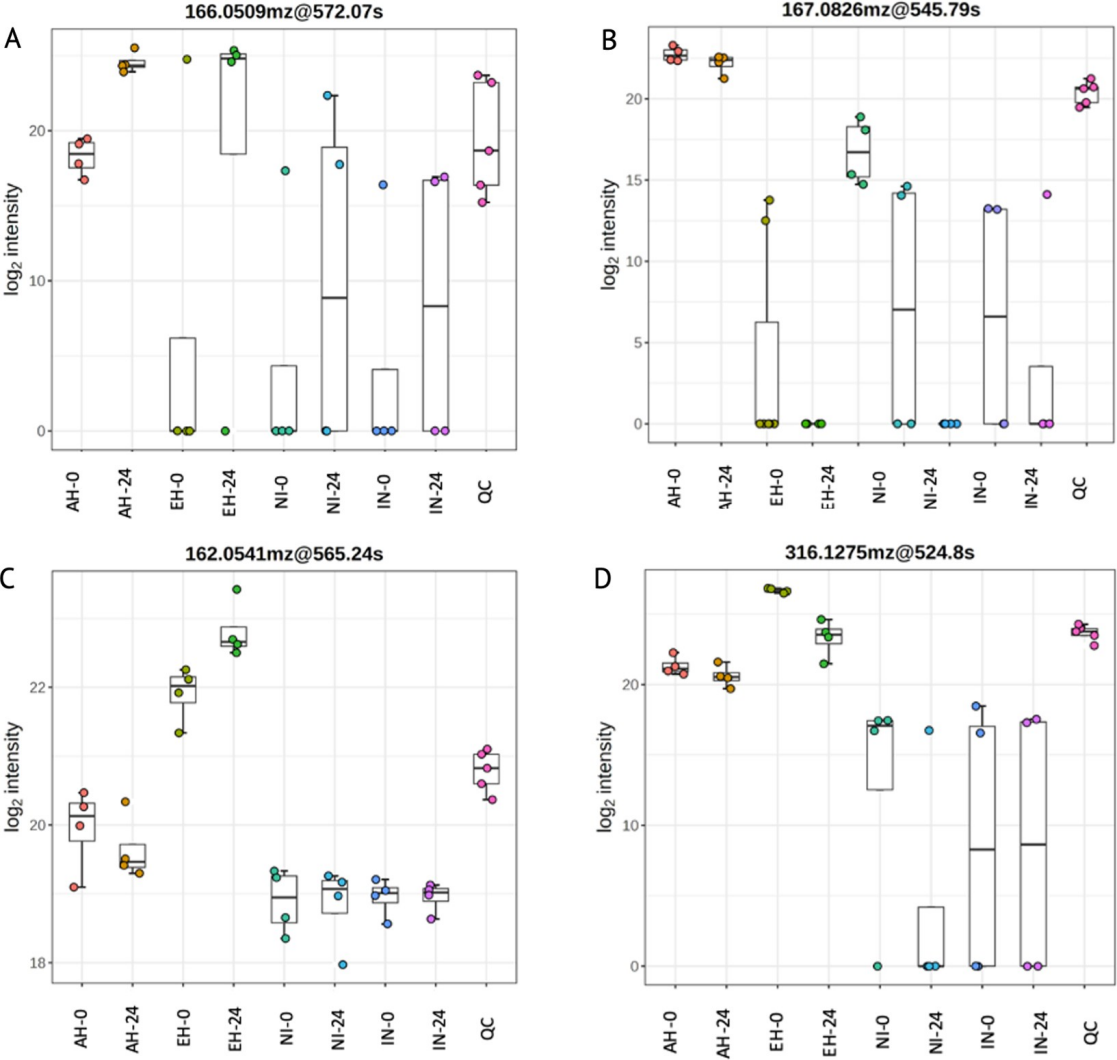
Significantly dysregulated metabolites associated with treatments as determined by putative identification within MetaboAnalyst 5.0. Vitamin B6 (a-b) and riboflavin (B2) (c-d)-associated metabolite abundance associated with each treatment at 0 and 24 h: Acid hydrolysate (AH-0, AH-24), enzyme hydrolysate (EH-0, EH-24), non-inoculated treatment (NI-0, NI-24), and inoculated treatment with *Campylobacter jejuni* (NI-0. NI-24). QC represents a positive control for mass spectrometry analysis and consists of the combination of all the samples. Pairwise comparisons indicated significant differences in metabolite abundance between AH and EH groups at 0 and 24 h time points (P < 0.05). Bar and whisker associated with the bar plots represent the mean and the standard deviation for each treatment.

**Fig 7:**
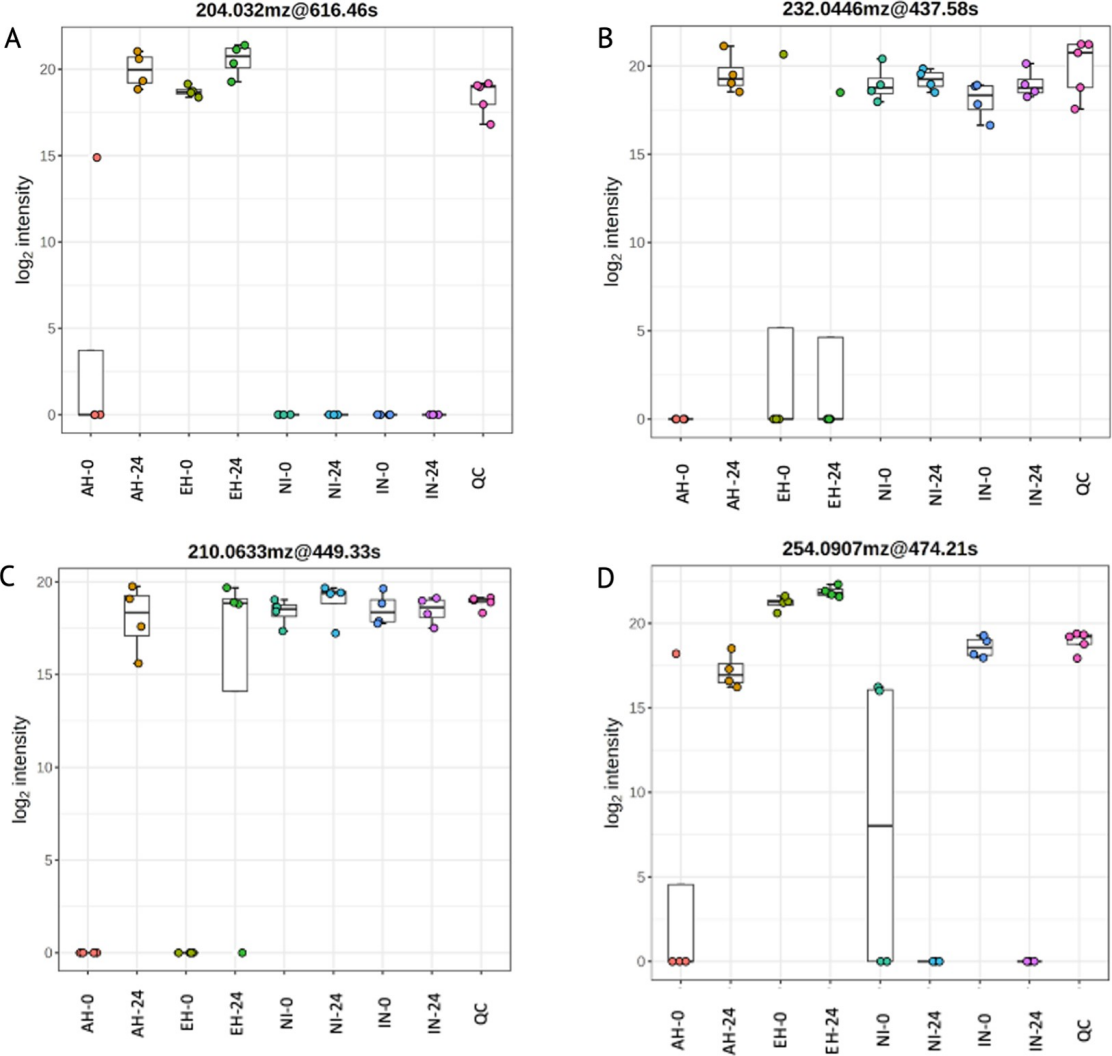
Significantly dysregulated metabolites associated with treatments as determined by putative identification within MetaboAnalyst 5.0. Dysregulated metabolites associated with folate metabolsim (a-d) for each treatment at 0 and 24 h: Acid hydrolysate (AH-0, AH-24), enzyme hydrolysate (EH-0, EH-24), non-inoculated treatment (NI-0, NI-24), and inoculated treatment with *C*. *jejuni* (IN-0, IN-24). QC represents a positive control for mass spectronomy analysis and consists of the combination of all the samples. Pairwise comparisons indicated significant differences in metabolite abundance between AH and EH groups at 0 and 24 h time points (P < 0.05). Bar and whisker associated with the bar plots represent the mean and the standard deviation for each treatment.

To ascertain the impact of casein supplementation on the functional aspects of the cecal system, we conducted functional analysis using MetaboAnalyst 5.0, employing the Mummichog algorithm and modified gene set enrichment analysis. Considering our focus on bacterial metabolism, we aligned the identified metabolites with potential pathways using the *Escherichia coli* library provided. The pathways that exhibited significant dysregulation are outlined in S10 Table. Vitamin B6 metabolism pathways (166.0509_572.07; 167.0826_545.79;) were significantly different for supplemented groups in the study (P < 0.05; Fig 6A and 6B), particularly AH and IN at 0 h; EH and IN at 0 h; AH at 24 h and AH and 0 h, EH at 24 and EH at 0 h, AH and EH at 24 h (S10 Table). Vitamin B6-associated metabolite 166.0509_572.07 increased for both EH and AH groups at 24 h (Fig 7A); whereas 167.0826_545.79 (Fig 6B) decreased in AH and increased in EH at 24 h. Riboflavin (vitamin B2)-associated metabolic pathways were also significantly affected by the treatments (P < 0.05; 162.0541_565.24; 316.1275_524.8; Fig 6C and 6D). At the 0-h mark, significantly different riboflavin-associated abundances were observed in AH and IN groups, as well as EH and IN groups. Furthermore, EH exhibited distinct riboflavin-associated abundances at both the 0-h and 24-h time points. Additionally, at the 24-h time point, significant differences in riboflavin-associated abundances were noted between AH and EH groups.

Vitamin B2-associated metabolites (162.0541_565.24) were increased in EH and decreased in AH at 24 h (Fig 6C); whereas 316.1275_524.8 were decreased in EH at 24 h but not significantly affected in abundance in AH group. The treatments produced significant differences in abundance of metabolites associated with folate (vitamin B9) biosynthesis (204.032_616.46; 232.0446_437.58; 210.0633_449.33; 254.0907_474.21; Fig 7), particularly AH and IN at 0h, EH and IN at 0h, AH at 24 h and AH at 0h, EH at 24 h and EH at 0h, and AH and EH at 24 h (S10 Table). Vitamin B9-associated metabolite 204.032_616.46 increased in abundance in AH and EH at 24 h; 232.0446_437.58 increased in abundance in AH at 24 h but was not significantly different in abundance in EH group at 24 h (Fig 7A); 210.0633_449.33 increased in abundance in 24 h in AH and EH groups (Fig 7B); 254.0907_474.21 increased in abundance in EH at 24 h (Fig 7C), but not as drastically as in AH group (Fig 7D). These results underscore the intricate interactions between peptide supplementation and the metabolism of essential B-vitamins within the cecal microbial community, shedding light on the nuanced dynamics of microbial communities in response to dietary interventions.

The significantly dysregulated annotated metabolites were aligned with the genomes of three taxa that exhibited differential core abundance at 24 and 48 h in EH and AH treatments: *Campylobacter*, *Faecalibacterium*, and *Phascolarctobacterium*. The selection of genomes was randomized, with species names chosen based on common members associated with poultry cecal environments. These annotated genomes comprised *C*. *jejuni jejuni* 51037, *F*. *prausnitzii* A2-165, and *P*. *faecium* JCM 30894, sourced from BioCyc [38]. The presence of high-abundance core microbiota and cecal microbial metabolic pathways acting on these dysregulated molecules suggests that these metabolites exert microbial effects on cecal community members. Therefore, this analysis aimed to assess overall cellular pathway changes linked to vitamin B metabolism, taking into account observed differences in genera core abundances between EH and AH treatments, along with vitamin B-associated metabolic variations (Figs 5–7). Additional vitamin B-associated pathways were impacted in three genera, including flavin, biotin, and folate metabolism (S11 Table). Specifically, the significantly differentiated metabolites were most perturbed in biotin biosynthesis from the 8-amino-7-oxononanoate I pathway and flavin biosynthesis I in *Campylobacter* and *Phascolarctobacterium* (S11 Table). Additionally, the biotin-carboxyl carrier protein assembly pathway was mapped to *Phascolarctobacterium*, while the flavin salvage pathway was associated with metabolites mapped to *Faecalibacterium*. Furthermore, folate pathways linked to the identified metabolites differed between *Campylobacter* and the two other genera, with the superpathway of tetrahydrofolate biosynthesis being expressed in *Campylobacter*. Folate transformations and folate polyglutamylation pathways were perturbed in Faecalibacterium and *Phascolarctobacterium*. Additionally, tetrahydrofolate biosynthesis II and tetrahydrofolate salvage from the 5,10-methenyltetrahydrofolate pathways were associated with *Phascolarctobacterium*’s overall cellular metabolism. Mapping metaboiltes to three bacterial genera revealed significant disruptions in biotin, flavin, and folate pathways, indicating potential metabolic mechanisms underlying differences in bacterial abundances between EH and AH treatments that can be explored further.

## Discussion

### Microbial dynamics in response to casein hydrolysates: Interactions, diversity, and metabolomic signatures within poultry GIT

Gaining insights into how hydrolyzed casein proteins (peptides and amino acids) can influence the microbiota population profiles within poultry’s GIT may impact the growth of pathogens such as *Campylobacter*. Additionally, these insights can provide further insight for identifying dietary strategies for modulating *Campylobacter* proliferation. The respective availability of proteins, peptides, and amino acids originating from the animals’ diet can play a pivotal role in shaping the microbial composition within the GIT, consequently affecting nutrient digestion and the resulting microbial metabolomic profiles [38]. In the present study, *Bacteroides* and *Enterococcus* constituted a fundamental microbiota component across all treatment groups. This observation aligns with earlier research that has indicated the elevated proteolytic activity of these taxa [40,41]. Consequently, the association of these taxa with all treatment groups is not unexpected. However, these taxa may be associated with cross-feeding interactions among microbial communities that are pivotal for maintaining system homeostasis.

An illustrative example is the *Bacteroides*-associated fermentation of non-digestible carbohydrates, which yields short-chain fatty acids (SCFA), CO_2_, and H_2_ [42]. Importantly, these gases are stabilized within the community through mutual cross-feeding, as other community members consume CO_2_ and H_2_ generated by *Bacteroides* [42]. Interestingly, *C*. *jejuni* does possess a hydrogenase enzyme that can oxidize H_2_, allowing it to grow beyond its microaerophilic limits [43,44]. Carbon dioxide can also be utilized by *C*. *jejuni*, as many *C*. *jejuni* strains require 1 to 10% of CO_2_ for growth [45]. This evidence suggests that *C*. *jejuni*’s survival and growth may benefit from cross-feeding interactions with other microbial members, particularly through the utilization of fermentation byproducts, such as CO_2_ and H_2_, generated by its fellow community members. Patuzzi et al. uncovered a positive correlation between the presence of Bacteroidales and *Campylobacter*-positive birds, while Clostridiales were associated with *Campylobacter*-negative birds, collectively comprising 80 to 90% of the total cecal microbial community [46]. Although the *C*. *jejuni* inoculum in the current study did not significantly increase microbial richness in the absence of protein supplementation, it is worth noting that *Campylobacter* abundance was highest in the EH group compared to the other treatment groups. Patuzzi et al. observed a significant impact on microbial richness with *Campylobacter* colonization, which varied depending on the timing of infection [46]. The intricate interactions within the microbiota highlight the complexity and adaptability of microbial communities within the gut ecosystem. These interactions can be influenced by the dietary composition and the resulting metabolic end products, which, in turn, may impact the environment of the system, such as pH levels.

Martinez-Lopez et al. demonstrated a higher Shannon diversity index in dogs fed a hydrolyzed protein diet than a high-protein diet [4]. This observation underscores the importance of the molecular weight and size of dietary proteins and peptides in influencing microbial community structure. Similarly, our current study investigated the effects of an EH and AH treatment on poultry cecal microbiota. We found that the EH treatment resulted in a higher mean Shannon diversity index than the IC treatment, with values of 4.2 in AH, 3.7 in EH, and 3.5 in IC. Moreover, our core microbiota analysis showed fewer IC-associated core microbial members at 48 h than the AH and EH groups. At 48 hours, we identified eight core microbial members associated with the IC treatment, while the EH and AH treatments exhibited 11 core members. This finding aligns with Martinez-Lopez et al. observations, suggesting that dietary components with lower molecular weights can contribute to greater microbial diversity [4].

In our study, to characterize the overall function of the cecal community, we mapped the identified metabolites to the *E*. *coli* to represent the pathways associated with the microbial community in poultry ceca. *E*. *coli* is widely studied as a prokaryotic model organism and plays a significant role in the cecal community, closely linked to *Campylobacter* colonization [47–49]. Our study has unveiled disparities in protein order of structure and molecular size, indicating differences among the core microbiota and the abundance of vitamin B-associated metabolites. For example, *Bacteroides* and *Ruminococcus*, capable of vitamin B2 production [50], were consistently identified as core taxa at 24 h in all treatment groups, except for NI and IC at 48 h. Riboflavin plays a pivotal role as an oxidation-reduction mediator and stimulates the growth of auxotrophic bacteria, such as *Faecalibacterium* [51]. An intriguing observation emerged regarding the core microbiota associated with EH and AH at 24 h, where nine core taxa were identical (Fig 5; S7 Table). The remaining differentiated taxa were *Phascolarctobacterium*, linked with EH treatment, and *Faecalibacterium*, associated with AH treatment. This distinction may hold significance considering our B vitamin-associated metabolism findings (S10 and S11 Tables). At the 24-h time point, the EH treatment demonstrated enhanced metabolic processes compared to the AH treatment. It is reasonable to speculate that these specific taxa may be connected to the observed variations in metabolomic pathways related to vitamin B6 and B9 metabolism. Prior research has suggested that *Faecalibacterium* does not produce vitamins B6 and B9, while *Phascolarctobacterium* does [52,53]. Thus, the presence or absence of these taxa within the core microbiota could play a pivotal role in influencing the metabolic profiles associated with these vital vitamins.

### Decoding *C*. *jejuni* metabolic strategies: Vitamin B dynamics and microbial interactions in the cecal system

In contrast to other pathogens, such as *Salmonella* that can alter the structure of the cecal microbial community and metabolome, as demonstrated in prior research by Mon et al. (2020), our current study found that *C*. *jejuni* did not exert a significant impact on the composition of the microbial community or the metabolomic profile within the cecal system, particularly in the absence of supplemented EH. Compared to the *Salmonella* genome of 5.1 million base pairs, the *C*. *jejuni* genome is only slightly over 1.5 million base pairs in size [54,55]. Potentially, the smaller genome of *C*. *jejuni* indicates a metabolic dependency on the products made by other microbial members in the local community, such as the cecal ecosystem. The cross-feeding interactions between the microbial community and *C*. *jejuni* possibly play a crucial role in the survival and persistence of *Campylobacter* within the system [9,55].

This cross-feeding dependency between *C*. *jejuni* and the local cecal microbiota may be rooted in their molecular preference for peptides over amino acids and intact casein and the concurrent production of vitamin B compounds within the system. When comparing the expression of B vitamin-associated metabolites among different treatments at each time point in the current study, we can postulate that these metabolic changes are a result of the *Campylobacter*-associated metabolism in relation to cecal microbiota based on treatment. Due to the 24 h preadaptation phase, during which the cecal community remained metabolically active and received fresh *C*. *jejuni* inoculations, we can reasonably conclude that the initial presence of *C*. *jejuni* at time 0 h did not have a substantial impact on the metabolic profile. Therefore, the metabolism of *C*. *jejuni*, based on the treatments applied, may have affected the metabolomic profiles associated with B vitamin metabolism at 24 h (Fig 7). Moreover, no significant dysregulation of metabolites was observed between the NI and IN groups at 24 h, indicating that the supplementation with EH had a pronounced effect on *Campylobacter*.

Various vitamin B6-associated metabolites (166.0509_572.07; 167.0826_545.79) exhibited differential abundance patterns for both AH and EH treatments (P < 0.05; Fig 6A and 6B). However, the EH treatment showed a considerably more pronounced change in metabolite abundance compared to the AH group. Notably, in the EH group, vitamin B6-associated metabolites were nearly undetectable at 0 h but showed a significant increase in abundance at 24 h (Fig 6B). *Campylobacter* species have been shown to synthesize vitamin B6, and it is possible that the utilization of EH sources, rather than amino acids, may enhance *C*. *jejuni’*s ability to synthesize vitamin B6. Asakura et al. established a connection between vitamin B6-dependent enzymes and flagellar glycosylation in *C*. *jejuni* [56]. Likewise, *Campylobacter*-related bacteria, such as *Helicobacter pylori*, have demonstrated a reliance on vitamin B6 for motility and virulence [57].

When examining riboflavin metabolism, it is worth noting that the AH treatment led to a decrease in riboflavin-associated metabolites. In contrast, there was a significant increase in the abundance of the same metabolites in the EH treatment (Fig 6C). Possibly, *C*. *jejuni* improved the abundance of riboflavin since *C*. *jejuni* was most abundant in the EH group and can internally synthesize vitamin B2 or assimilate it from metabolites present in the host environment [58]. More so, the uptake of riboflavin by *C*. *jejuni* plays a role in ferritin reductase activity and iron assimilation, thereby contributing to bacterial pathogenicity. Additionally, pentose phosphate pathway and pyrimidine metabolism were increased in abundance in EH treatment at 24 h compared to 0 h, indicating potential biosynthesis of riboflavin and flavin adenine dinucleotide (FAD) and flavin mononucleotide (FMN) compounds (S10 and S11 Tables) [58]. Remarkably, in the EH treatment, riboflavin metabolism appears to have undergone a shift from one metabolite to another. Specifically, there was a significant increase in the abundance of one metabolite (162.0541_565.24) at 24 h, while another metabolite (316.1275_524.8) experienced a notable decrease in abundance during the same time frame (Fig 6C and 6D). Riboflavin metabolism was not significantly different for AH treatment for 0 and 24 h. These findings suggest that the presence of larger peptides in distal GIT compartments, such as the ceca, could potentially contribute to the prevalence, colonization, and virulence of *C*. *jejuni* by providing the pathogen with an additional source of energy production.

The biosynthesis of folate by GIT bacteria is influenced by a low-carbohydrate diet and an elevated protein content [59]. In our study, in contrast to the dysregulation observed in other B vitamin metabolites, the AH treatment group exhibited a remarkable increase in folate-associated metabolites at 24 h when compared to the EH treatment (204.032_616.46; 232.0446_437.58; 210.0633_449.33; 254.0907_474.21; Fig 7A–7D). Bai et al. observed that when laying hens were supplemented with folate, unabsorbed folate was detected in the lower GIT compartments, specifically the ceca [60]. Similar to the findings of the current study, while cecal microbial diversity remained unaffected, there was a negative correlation observed between folate supplementation and the presence of *Faecalibacterium* and *Campylobacter* [60]. In comparison, *Lactobacillus* and *Bifidobacterium* have been observed to be positively correlated with folate status [59,60]. This finding indicates that the use of proteases to decrease the abundance of larger peptides in the lower GIT compartments may improve the ratio of beneficial bacteria to potential pathogens, thus decreasing transmission of pathogens throughout the flock and improving poultry performance and production.

B vitamins play pivotal physiological roles within the host by supporting the fitness of symbiotic microorganisms while simultaneously inhibiting the proliferation of competitive species, as highlighted by Uebanso et al. [59]. Our study showed that *C*. *jejuni* inoculated EH resulted in a drastic increase in an abundance of *C*. *jejuni* (Fig 3E) and vitamin B6 (Fig 7A and 7B), compared to AH treatment. At the same time, vitamin B9-associated metabolites were drastically increased in the AH group from 0 to 24 h compared to EH treatment. Although dietary B vitamins are primarily absorbed in the small intestine, any excess B vitamins may find their way into the distal GIT compartments. Within these distal GIT compartments, B vitamins can function as nutrients for the local microbial inhabitants, thereby promoting the growth of beneficial bacteria and suppressing the colonization of pathogens. Hence, investigating the molecular preferences of *C*. *jejuni* and the local microbiota for peptides and amino acids is imperative. This understanding can help in the development of dietary interventions, such as the incorporation of exogenous enzymes aimed at supporting beneficial bacteria while concurrently starving pathogens like *Campylobacter*.

Furthermore, *Faecalibacterium* has been shown in multiple studies to engage in competitive interactions with *Campylobacter*. Our findings suggest potential competitive factors may involve vitamin B-associated metabolism in peptide-rich environments (S11 Table) [46]. Findings by Patuzzi et al. showed that *Campylobacter* does not interact positively with the cecal microbial community. However, *Campylobacter* accounted for 32 negative interactions with taxa, such as *Pseudomonadaceae*, *Parabacteroides*, and three other taxa that positively interacted with *Lactobacillus* and *Faecalibacterium*. Thus, *Campylobacter* can potentially suppress bacterial populations promoted by *Faecalibacterium* and *Lactobacillus* under suitable conditions. Moreover, *Campylobacter’s* capacity to synthesize B vitamin-associated metabolites grants a competitive advantage over *Faecalibacterium*, as our research findings indicate that potential competitive factors may involve metabolic pathways associated with vitamin B6 and B9.

### Supporting a healthy microbial barrier: Peptides as modulators of GIT microbial balance

Research conducted by Aloo and Oh underscores the ability of peptides to influence the microbial balance between beneficial microbiota and pathogens [3]. An intriguing observation was the presence of *Faecalibacterium* as a core microbiota member in the AH group at 48 h, while *C*. *jejuni* was notably absent from the core community at the same time point. Conversely, in the EH group, *Faecalibacterium* was not part of the core microbial community at 24 or 48 h, whereas *C*. *jejuni* was consistently present (Fig 5; S7 Table). Increased relative abundance of *Campylobacter* in EH treatment may indicate a potential connection between the type of dietary protein and the prevalence of this pathogen, supporting the idea of Aloo and Oh on the ability of peptides to impact microbial compositions [3]. Furthermore, these findings support the intricate interplay occurring between *Faecalibacterium* and *Campylobacter* [46], which is influenced by the dietary environment.

In another study, a shift towards beneficial bacteria was noted using a protease. Hua et al. reported an increase in the relative abundance of *Lachnospiraceae*, *Bacteroides*, *Ruminococcaceae*, and various other beneficial bacteria in the rats’ GIT after administering a protease hydrolysate treatment [61]. The presence of a protease associated with protein breakdown could signify the existence of both larger and smaller peptides, as seen in the EH and AH groups utilized in the current study. Notably, the groups *Lachnospiraceae*, *Bacteroides*, and *Ruminococcus torques* persisted in the supplemented groups until 48 h, except for *the Ruminococcus torques group*, which was absent in the IC group at the 48-h time point. Additionally, Wang et al. showed that administering enzymatically hydrolyzed collagen peptide in mice decreased the abundance of bacteria associated with GIT inflammation, such as *Erysipelatoclostridium* [62]. In the current study, the abundance of *Erysipelatoclostridium* decreased by 27% (ANCOM, P < 0.05) with time across treatments. Also, *Erysipelatoclostridium* was a core microbial community member in EH at 0 h and was absent at 24 and 48 h in all treatments. As a member of the *Clostridia* class, *Erysipelatoclostridium* is known for its ability to ferment amino acids through coupled reduction and oxidation of amino acids to organic acids [63]. This characteristic may explain this genus’s rapid decline in the EH group, suggesting its inability to utilize peptides for growth. While not all Clostridial members are pathogenic, it is essential to note that commensal *Clostridia* play a crucial role in improving GIT function, as demonstrated by Lopetuso et al. [64]. Maintaining an abundance of resident *Clostridia* species in the poultry GIT, possibly achieved through the utilization of proteases in the diet or hydrolyzed proteins to increase digestion rates in the upper GIT compartments while delivering smaller protein particles into the lower compartments, could potentially help reduce *Campylobacter* abundance in the ceca. This reduction could potentially be attributed to *Campylobacter*’s preference for peptides over amino acids and polypeptides, as observed in this study, while promoting the local growth of *Clostridia* in the lower GIT compartments, such as the ceca.

Enhancing protein digestion in the upper GIT compartments and reducing the presence of larger peptides in the lower GIT, achieved by using proteases and protein hydrolysates, can also induce a tolerogenic immune response that promotes systemic anti-inflammation [42]. For instance, the administration of casein glycomacropeptide hydrolysate (CGP) has demonstrated beneficial effects in diabetic mice by modulating the GIT microbiota [65]. GGP supplementation significantly reversed the abundance of *Bacteroides* and *Proteobacteria*, leading to an increased *Bacteroides* to *Firmicutes* ratio and an elevated abundance of *Ruminiclostridium*, *Allobaculum*, and *Blautia*. Simultaneously, it reduced the abundance of Helicobacteriacea compared to the type 2 diabetes-induced group. Notably, these microbial taxa can produce short-chain fatty acids (SCFA), which enter the systemic circulation and possess anti-inflammatory properties [42]. The decrease in pathogenic taxa such as Helicobacteriacea is a promising indicator of the positive impact of peptides on the microbiota ratios within the GIT. Incorporating exogenous enzymes and enzyme hydrolysates in poultry feed can be advantageous, particularly when supplemented with various polysaccharides. By utilizing the Maillard reaction, this combination can facilitate molecular rearrangement and modification, producing new, smaller molecules that enhance the stability and bioactivity of peptides [14]. In a study by Zhang et al., soybean peptides, L-cysteine, and xylose were combined and subjected to the Maillard reaction at 140°C for 2 hours [56]. The administration of these reaction products into the diets of mice demonstrated an improvement in the abundance of SCFA-producing bacteria, such as *Bifidobacterium* and *Lactobacillus*.

## Conclusions

While the current study acknowledges experimental limitations, such as the absence of non—*C*. *jejuni* inoculated casein supplemented groups and being focused on cecal incubations only to the exclusion of the upper GIT, it does offer insight into the direct impact of different casein supplements on cecal microbial populations inoculated with *C*. *jejuni*, aiming to model the natural ecology of the cecal system that would potentially contain a wide range of protein and corresponding hydrolysate products. The chosen media supports a diverse range of microbial members, including *Campylobacter*, enabling a comprehensive comparison of all supplemented groups against the non-supplemented treatment with *C*. *jejuni* inocula. Additionally, the non-inoculated group and inoculated groups without casein supplementation provide insights into microbial dynamics solely based on the presence of *C*. *jejuni* within the cecal system. More specifically it appears that the form of casein supplementation impacts *C*. *jejuni* prevalence in the ceca and may serve to enrich for this pathogen in the presence of other cecal microbiota.

Our study suggests that there is complex interplay between dietary protein composition and the *C*. *jejuni* inoculated poultry cecal microbiota inoculated with *C*. *jejuni* and the corresponding metabolomic profiles within the *in vitro* poultry cecal system. Our study suggests that there is complex interplay between dietary protein composition and the *C*. *jejuni* inoculated poultry cecal microbiota inoculated with *C*. *jejuni* and the corresponding metabolomic profiles within the *in vitro* poultry cecal system. Microbial communities inoculated with *C*. *jejuni* and the metabolomic profiles within the *in vitro* poultry cecal system. *C*. *jejuni* did not significantly alter the composition of the cecal microbial community or metabolomic profile without supplemented peptides. Indeed, casein supplementation improved microbial evenness and richness compared to non-supplemented groups. The abundance of *C*. *jejuni* was increased in the casein enzyme hydrolysate group, indicating a preference for peptides over amino acids and intact casein in the current study. The potential cross-feeding interactions during casein modifications may be based on B vitamin-associated metabolism. The study highlighted several noteworthy findings regarding the impact of different treatments on B vitamin metabolism. The presence of *C*. *jejuni* in enzyme hydrolysate treatment notably influenced the abundance of vitamin B6 and B9-associated metabolites. Moreover, whether hydrolyzed or not, the type of dietary protein significantly impacted microbial diversity, with hydrolyzed protein treatments resulting in higher diversity. The presence of specific core microbial taxa, such as *Bacteroides*, *Ruminococcus*, *Faecalibacterium*, and *Phascolarctobacterium*, appeared to play a role in shaping the metabolic profiles associated with B vitamin metabolism. These findings emphasize the intricate relationship between the GIT ecosystem’s dietary components, microbial communities, and metabolic pathways.

Furthermore, our findings corroborate earlier research regarding the modulation of microbial ratios through the utilization of hydrolyzed proteins, promoting the growth of beneficial bacteria while diminishing the prevalence of potential pathogens. The significant increase in *Campylobacter* abundance noted in the enzyme hydrolysate group suggests that a decrease in peptide presence in the lower GIT compartments, such as poultry ceca, could potentially diminish *Campylobacter* prevalence. However, this observation warrants further validation through in vivo studies that integrate host factors and relevant protein digestion mechanisms based on endogenous processes. Any reduction would likely manifest as a decrease in *Campylobacter* abundance in fecal and cecal droppings. Understanding these interactions holds promise for developing strategies to control pathogens such as *Campylobacter* while promoting beneficial microbial populations, ultimately improving animal health and food safety in the poultry industry [60].

## Supporting information

S1 Table Feed ingredient composition and calculated nutrient content of basal diets. ^1^ Premix provided the following ingredients per kilogram of diet: Copper, 15 mg; iron, 40 mg; zinc, 100 mg; manganese, 100mg; selenium, 0.35 mg; iodine, 1 mg; vitamin A, 10,000 IU; vitamin D3, 5,000 IU; vitamin E, 80 IU; vitamin K, 3 mg; vitamin B_1_, 3 mg; vitamin B_2_, 9 mg; vitamin B_6_, 4 mg; vitamin B_12_, 0.02 mg; nicotinic acid, 60 mg; pantothenic acid, 15 mg; biotin, 0.15 mg; folic acid, 2 mg. ^2^ Diets were supplemented with 0.75% sand (Control), 0.20% PB and 0.55% sand, 0.50% PB and 0.20% sand, or 0.75% PB. ^3^ Nutrient contents were calculated on a dry matter basis.
(XLSX)

S2 Table Interactions based on alpha diversity metrics via ANOVA, where the effect of treatment (peptide modifications: Intact casein, enzyme hydrolysate, and acid hydrolysate and *Campylobacter jejuni* inoculation) was assessed on microbial diversity based on time (0, 24, and 48 h). Shannon’s entropy was utilized to assess richness of microbial composition and Pielou’s Evenness was used to describe homogeneity of microbial community. Significance was determined at P < 0.05. *Sums of Squares. *Projected Residuals: Fit values for metric vs. observation error (residuals). Residuals represent the difference between any data point and the regression line.
(XLSX)

S3 Table Interactions based on beta-diversity metrics via ADONIS, where effect of treatment (peptide modifications: Intact casein, enzyme hydrolysate, and acid hydrolysate and *Campylobacter jejuni* inoculation) was assessed on microbial composition based on time (0, 24, and 48 h). Bray-Curtis was utilized to describe abundance between the samples and Weighted Unifrac was used to assess phylogenetic differences between microbial communities. Significance was determined at P < 0.05. *Sums of Squares. *Mean Squares. *Projected Residuals: Fit values for metric vs. observation error (residuals). Residuals represent the difference between any data point and the regression line.
(XLSX)

S4 Table Kruskal-Wallis results for pairwise comparison between treatment groups. 1Bolded values are those with significant p and q values. 2H value is the test statistic for the Kruskal-Wallis test. A sufficiently high test statistic indicates that at least one difference between the medians is statistically significant.
(XLSX)

S5 Table ANOSIM pairwise results for the beta diversity metrics for treatments used in the current study. ^1^Bolded values are those with significant p and q values. ^2^R is the ANOSIM statistic, compares the mean of ranked dissimilarities between groups to the mean of ranked dissimilarities within groups.
(XLSX)

S6 Table Taxonomic diversity associated with five different treatments (Non-inoculated, Inoculated, Intact casein, Enzyme hydrolysate, and Acid hydrolysate) at three time points (0, 24, and 48) utilized in the current study. (XLSX)

S7 Table Core microbiota associated with each treatment at three time points (0, 24, and 48 h) used in the current study. (XLSX)

S8 Table ANCOM results and percentile abundance for the significantly differentiated taxon associated with five treatments used in the current study. (XLSX)

S9 Table Feature table of metabolites from liquid chromatography—mass spectrometry (LC-MS) analysis. MS1 spectra matched to spectra and compounds in Human Metabolome Database using MetaboAnalyst 5.0.
(XLSX)

S10 Table Functional analysis of dysregulated metabolites associated with treatments in the current study. Pathway analysis results derived from functional analysis module in MetaboAnalyst 5.0 using Mummichog 2.0 (P-value < 0.00001, KEGG Escherichia coli library).
(XLSX)

S11 Table Top 50 pathways based on perturbation scores (PPS) that were assessed via annotated metabolites mapped to *Phascolarctobacterium faecium*, *Campylobacter jejuni*, and *Faecalibacterium prausnitizii* genomes in BioCyc. (XLSX)
